# Canine Butterfly Glioblastomas: A Neuroradiological Review

**DOI:** 10.3389/fvets.2016.00040

**Published:** 2016-05-19

**Authors:** John H. Rossmeisl, Kemba Clapp, Theresa E. Pancotto, Samantha Emch, John L. Robertson, Waldemar Debinski

**Affiliations:** ^1^Veterinary and Comparative Neuro-Oncology Laboratory, Virginia Maryland College of Veterinary Medicine, Virginia Tech, Blacksburg, VA, USA; ^2^Department of Small Animal Clinical Sciences, Virginia Maryland College of Veterinary Medicine, Virginia Tech, Blacksburg, VA, USA; ^3^Department of Biomedical Engineering and Mechanics, Virginia-Tech Wake Forest School of Biomedical Engineering, Blacksburg, VA, USA; ^4^Comprehensive Cancer Center and Brain Tumor Center of Excellence, School of Medicine, Wake Forest University, Winston-Salem, NC, USA; ^5^Department of Neurology and Neurosurgery, VCA Alameda East Veterinary Hospital, Denver, CO, USA

**Keywords:** astrocytoma, brain tumor, canine, glioblastoma, magnetic resonance imaging

## Abstract

In humans, high-grade gliomas may infiltrate across the corpus callosum resulting in bihemispheric lesions that may have symmetrical, winged-like appearances. This particular tumor manifestation has been coined a “butterfly” glioma (BG). While canine and human gliomas share many neuroradiological and pathological features, the BG morphology has not been previously reported in dogs. Here, we describe the magnetic resonance imaging (MRI) characteristics of BG in three dogs and review the potential differential diagnoses based on neuroimaging findings. All dogs presented for generalized seizures and interictal neurological deficits referable to multifocal or diffuse forebrain disease. MRI examinations revealed asymmetrical (2/3) or symmetrical (1/3), bihemispheric intra-axial mass lesions that predominantly affected the frontoparietal lobes that were associated with extensive perilesional edema, and involvement of the corpus callosum. The masses displayed heterogeneous T1, T2, and fluid-attenuated inversion recovery signal intensities, variable contrast enhancement (2/3), and mass effect. All tumors demonstrated classical histopathological features of glioblastoma multiforme (GBM), including glial cell pseudopalisading, serpentine necrosis, microvascular proliferation as well as invasion of the corpus callosum by neoplastic astrocytes. Although rare, GBM should be considered a differential diagnosis in dogs with an MRI evidence of asymmetric or symmetric bilateral, intra-axial cerebral mass lesions with signal characteristics compatible with glioma.

## Introduction

**Figure 1:**
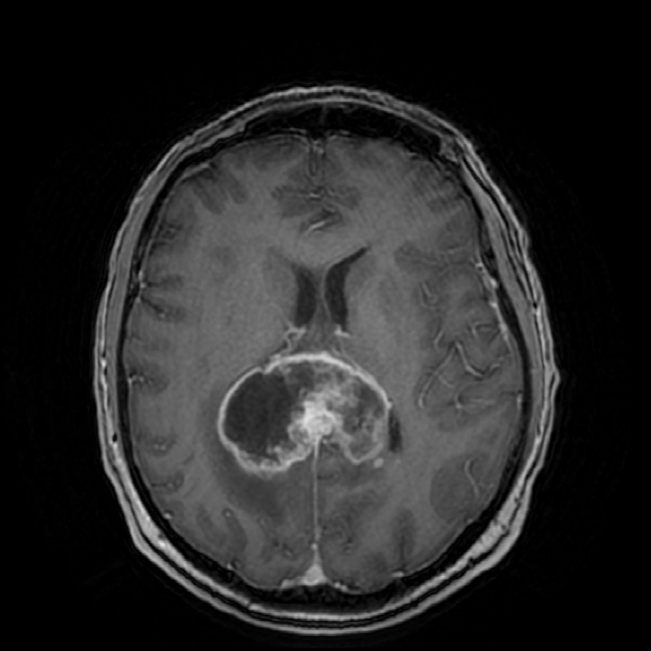
**Coronal, post-contrast T1 image of a posterior human butterfly GBM**.

The term “butterfly glioma (BG)” is used as a morphologic descriptor for human high-grade astrocytomas that extend across midline *via* white matter commissures, resulting in a contiguous bihemispheric lesion (Figure 1) whose wing-like extensions may display mirror image symmetry (1, 2). The most common manifestation of a BG is bifrontal glioblastoma multiforme (GBM), with invasion of the contralateral hemisphere occurring *via* the genu of the corpus callosum (1, 2). Anaplastic astrocytomas have also been associated with BG appearances, and bihemispheric spread of high-grade astrocytomas may also occur *via* other portions of the corpus callosum, including the body and splenium (1). Glial tumors account for approximately 35% of all primary intracranial neoplasms in dogs, and numerous studies have demonstrated the neuropathological and magnetic resonance imaging (MRI) homologies that exist between canine and human gliomas (3–8). However, to our knowledge, canine gliomas displaying neuroradiological or pathological features consistent with BG have not been reported.

## Case Presentations

### Case 1

A 13-year-old, spayed female mixed-breed dog presented with a 10-day history of intermittent aggression and circling to the left. A cluster of three generalized seizures occurred the evening prior to evaluation. Neurological examination abnormalities noted at admission included obtundation, propulsive circling to the left, a left head turn, absent menace responses OU, diminished visual tracking OU, normal pupillary light reflexes OU, and postural reaction deficits in the right thoracic and both pelvic limbs. The neuroanatomic diagnosis was a multifocal or diffuse prosencephalic lesion that was suspected to be more severe on the left side. An MRI examination of the head was obtained under general anesthesia (Supplementary Material).

**Figure 2:**
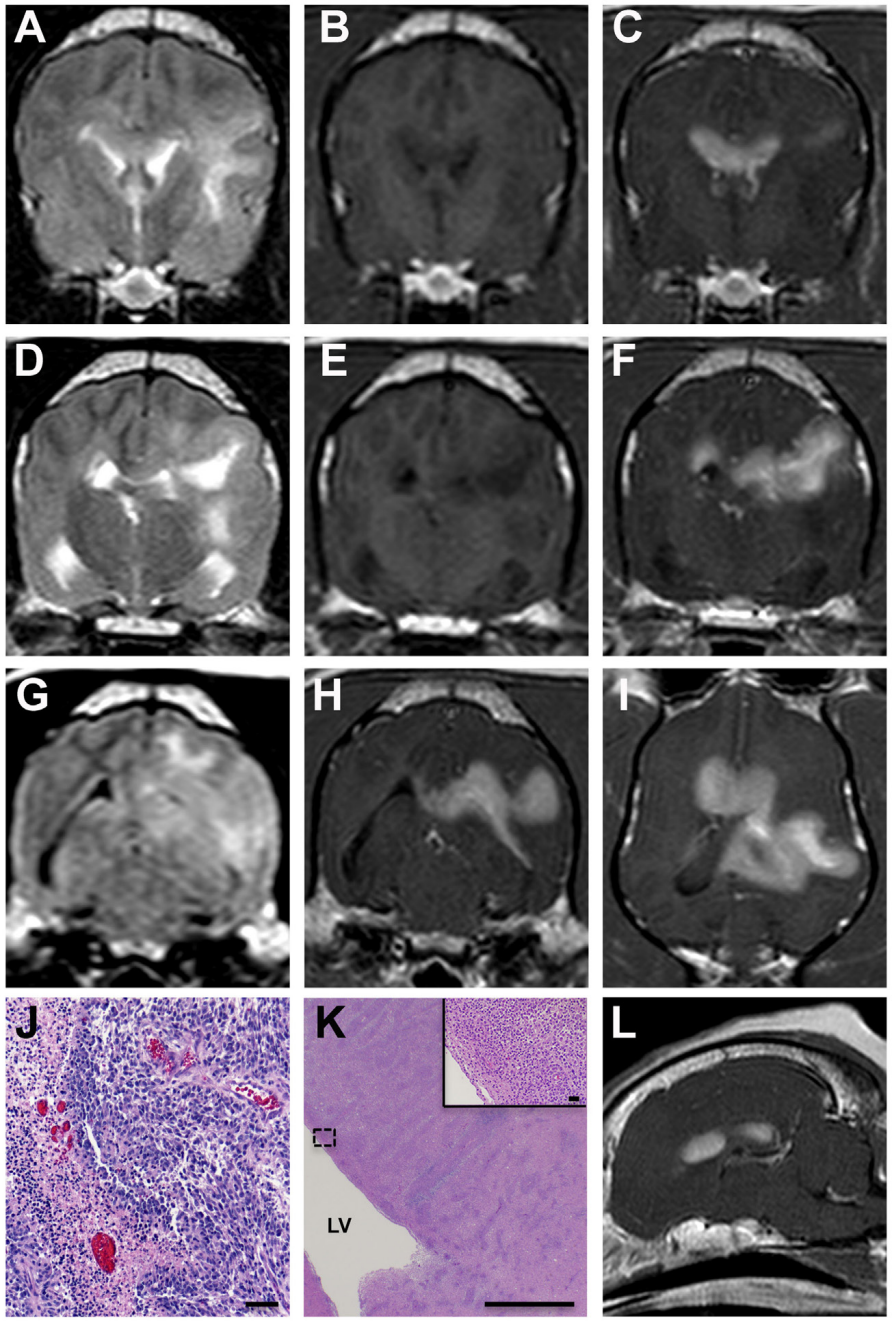
**MRI and pathological features of asymmetrical butterfly glioblastoma, Case 1**. Transverse T2 **(A,D)**, T1 **(B,E)**, FLAIR **(G)**, and post-contrast T1 **(C,F,H)** MR images from the rostral aspect of the intra-axial mass at the level of the cruciate gyrus **(A–C)**, the midportion of the lesion **(D–F)**, and caudal aspects of the mass at the level of the mesencephalic aqueduct **(G,H)**. The mass is T1 hypo- to isointense, T2/FLAIR hyperintense and demonstrates marked contrast enhancement. Mass effect, manifest as left lateral ventricular compression and a falx shift to the right, and T2/FLAIR hyperintensity in the perilesional white matter are evident. The extensive corpus callosal and bihemispheric involvement of the contrast-enhancing lesion burden is depicted in the T1 post-contrast dorsal planar **(I)**, transverse **(C,F,H)**, and parasagittal images **(L)**. Microscopic features of GBM **(J)** include serpentine necrosis, glial pseudopalisading, and microvascular proliferation, H&E stain, bar = 125 μm. Diffuse neoplastic infiltration of the periventricular white matter [**(K)**; LV, right lateral ventricle; bar = 3.5 mm] of the right cerebrum, H&E stain, bar = 40 μm, inset.

Tracking throughout the white matter of the left parietal lobe was a markedly and homogeneously contrast-enhancing, T2 hyperintense and T1 hypointense intra-axial mass with lobulated margins that measured 2.4 cm × 2.9 cm × 2.2 cm at its largest dimensions (Figures 2A–I,L). The mass extended caudally along the margin of the left lateral ventricle and crossed midline along the dorsal and rostral aspects of the corpus callosum. There was no evidence of fluid-attenuated inversion recovery (FLAIR) suppression or GRE signal void in the lesion.

Following completion of the MRI examination, the dog was euthanized at the owner’s request, and a necropsy was performed. On transverse sections of the brain, there was a large tan–gray mass centered in the left subcortical white matter extending from the frontal lobe caudally to the occipital lobe. In the left parietal lobe, the tumor extended dorsolaterally into the meninges and obliterated the normal cortical architecture.

As the microscopic features of the neoplasms were similar between all cases, they are described in detail for Case 1 as follows, with the other case descriptions highlighting differences between the tumors. The mass predominantly comprised a pleomorphic population of neoplastic astrocytes admixed among serpentine regions of necrosis. Most necrotic regions were surrounded by smaller fusiform-shaped glial cells arranged in a pseudopalisading pattern (Figure 2J). The periphery of necrotic areas contained multifocal areas of microvascular proliferation, throughout which were scattered hemorrhagic foci. The majority of neoplastic cells had ovoid to elongate nuclei but demonstrated marked heterogeneity, variable density, and significant anisokaryosis. The mitotic rate was 8–10/40× field, and mitotic figures displayed both normal and atypical morphologies. Neoplastic cells invaded extensively into the subependymal region of the left lateral ventricle through the subcortical white matter of the left cerebral hemisphere, the corpus callosum, and extended into the corona radiata and internal capsule of the right cerebral hemisphere (Figure 2K). Neoplastic cells demonstrated highly and regionally variable immunoreactivity to both GFAP and vimentin. The final diagnosis was glioblastoma with bihemispheric involvement.

### Case 2

A 6-year-old spayed female Beagle was evaluated for a 1-week history of abnormal behaviors, including head pressing and propulsive bidirectional circling interspersed between periods of somnolence. The dog had also experienced at least one generalized seizure during this period. Upon examination, the dog’s systolic blood pressure was 160 mmHg, and its small animal coma scale score (SACS) was 12/18, characterized by lateral recumbency with intermittent opisthotonus, bilateral unresponsive miosis, and periods of stupor characterized by constant vocalization. A diffuse or multifocal forebrain disorder was suspected. A brain MRI examination was performed under general anesthesia.

**Figure 3:**
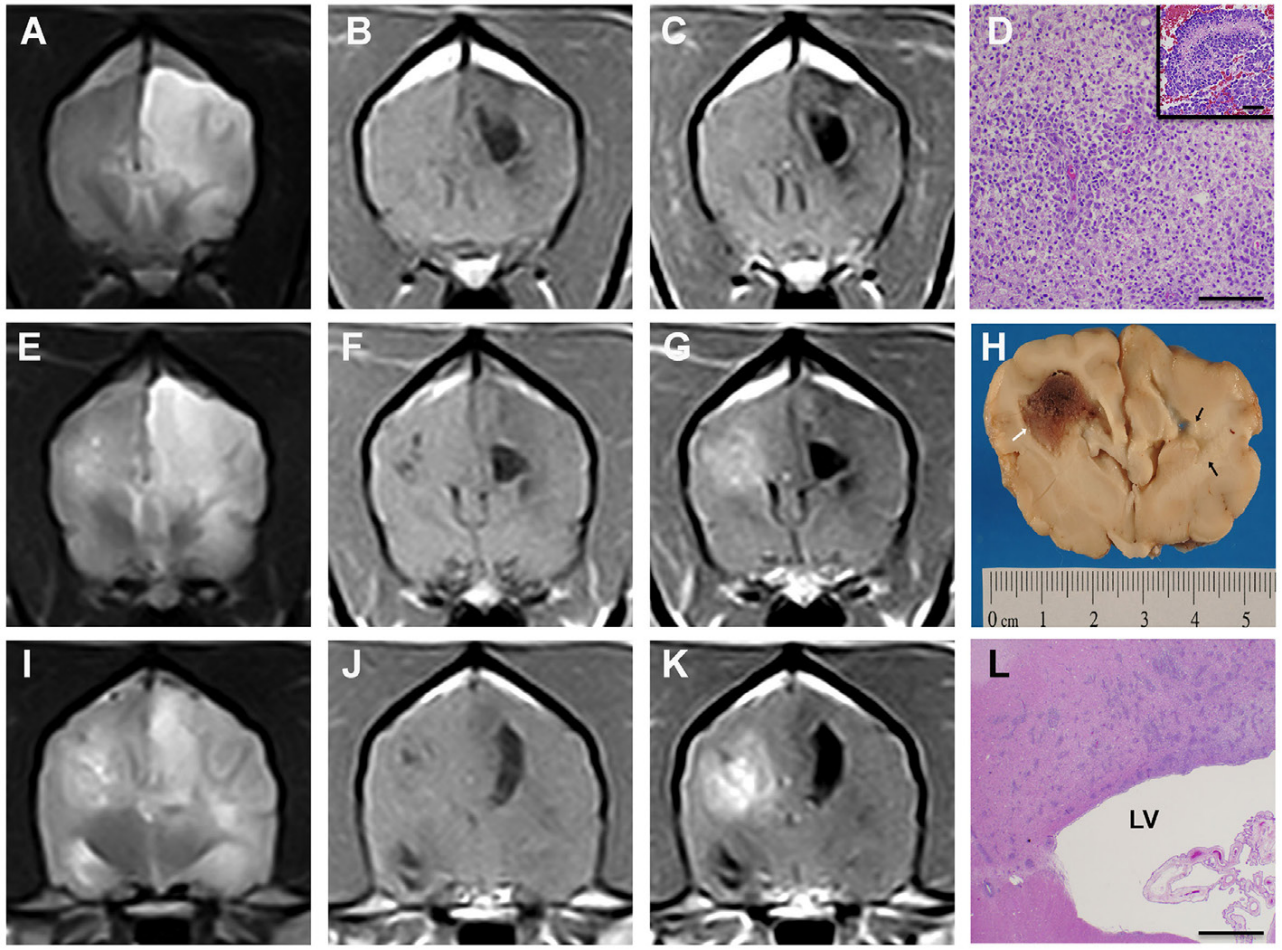
**Asymmetrical butterfly glioblastoma, Case 2**. Transverse T2 **(A,E,I)**, T1 **(B,F,J)**, and post-contrast T1 **(C,G,K)** MR images from the rostral aspect of the left cerebral lesion **(A–C)**, the midportion of the lesions **(E–G)**, and caudal portions of the mass lesions **(I–K)**. The masses are heterogeneously T1 hypo- to isointense, T2 hyperintense, and the right cerebral lesion demonstrates heterogeneous contrast enhancement. In the rostral aspect of the lesion, T2 hyperintensity extending from the left cerebral mass lesion across midline **(A)** *via* the corpus callosum is evident, which was demonstrated to be caused by neoplastic invasion of the corpus callosum at necropsy [**(D)**, H&E, bar = 500 μm]. Morphology of GBM [**(D)**, inset] within right cerebral mass illustrating serpentine necrosis and hemorrhage, H&E, bar = 75 μm. Bilateral mass effect, manifest as lateral ventricular compression and a falx shift to the right is present. Gross brain specimen **(H)** at the level of the optic chiasm demonstrating mass lesion in right cerebrum (white arrow) and malacia of the subcortical white matter on the left (black arrows). At the caudal level, the left and right intra-axial mass lesions are connected *via* T2 hyperintensity **(I)** extending across corpus callosum. Photomicrograph **(L)** of multifocal infiltration of neoplastic astrocytes within the body of the corpus callosum, H&E stain, bar = 3 mm, LV, left lateral ventricle.

On MRI images, two distinct intra-axial lesions were seen in the cerebral hemispheres (Figure 3). On the left was a highly infiltrative, non-contrast-enhancing, T2 hyperintense and T1 hypointense lesion that encompassed the frontal, parietal, and occipital lobes, causing indistinct margins between the gray and white matter (Figures 3A–C,E–G,I–K). Dorsally in the parietal lobe, immediately adjacent to the left lateral ventricle, was a T2 hyperintense, T1 hypointense, and FLAIR suppressing lesion that measured 1.6 cm × 0.7 cm × 1.1 cm and likely represented a cystic component of the mass. In the right parieto-occipital region was a well-circumscribed, T2 hyperintense and T1 isointense mass that moderately contrast enhanced and measured 1.4 cm in diameter (Figures 3E–G,I–K). Scattered throughout the right-sided mass were pockets of T2 hyperintense, T1 hypointense, and FLAIR suppressing material, likely representing cystic areas similar to the lesion on the left. At the most caudal lesion level, T2 hyperintensity within the corpus callosum was contiguous with left and right cerebral mass lesions (Figure 3I). There was no evidence of GRE signal void throughout either side of the cerebrum. Deviation of the falx cerebri and displacement of the caudoventral aspect of the cerebellum through the foramen magnum were present.

Following the MRI examination, the dog’s SACS deteriorated to 9/18, and there was no clinical improvement following administration of mannitol (1 g/kg IV), furosemide (1 mg/kg IV), or dexamethasone (0.25 mg/kg IV q 12 h). Approximately 30 h after admission, the dog experienced cardiopulmonary arrest. A necropsy was performed.

On gross examination, the external surface of right frontal cerebral cortex appeared swollen, and herniation of the cerebellum through the foramen magnum was observed. During transverse sectioning of the brain, a reddish-brown intra-axial mass centered in the subcortical white matter was observed extending from the dorsal aspect of the right frontal lobe (Figure 3H) caudally to the occipital lobe. The right hemispheric mass caused attenuation of the right lateral ventricle and a deviation of the falx cerebri to the left of midline. Several large foci of coalescing malacia were apparent, involving the corpus callosum, and extending to into the corona radiata (Figure 3H) and internal capsule of the left cerebral hemisphere.

The right cerebral lesion was composed of a pleomorphic population of neoplastic astrocytes that infiltrated and crossed the corpus callosum (Figures 3D,L). Compared to Case 1, the mass lesion in the right cerebral hemisphere contained larger areas of necrosis and lesser microvascular proliferation. In the left cerebral hemisphere, large geographic areas of coagulative necrosis, numerous intravascular thrombi, and an accompanying marked gitter cell reaction with less prominent lymphocytic infiltrates were observed. In the left cerebrum at the peripheral interface between necrotic tissue and edematous white matter, there were regions of infiltrating neoplastic astrocytes. Approximately 25% of the neoplastic cells demonstrated GFAP immunoreactivity. The final diagnosis was glioblastoma with bilateral cerebral involvement.

### Case 3

A 7-year-old, castrated male Whippet presented with a 1-month history of visual dysfunction and generalized seizures, was evaluated. Neurological examination abnormalities observed were compatible with a diffuse prosencephalic disorder and included a pacing gait with mild generalized ataxia, proprioceptive deficits in all limbs, absent visual tracking OU, and absent menace responses OU associated with normal pupillary light reflexes and unremarkable ophthalmic examination.

**Figure 4:**
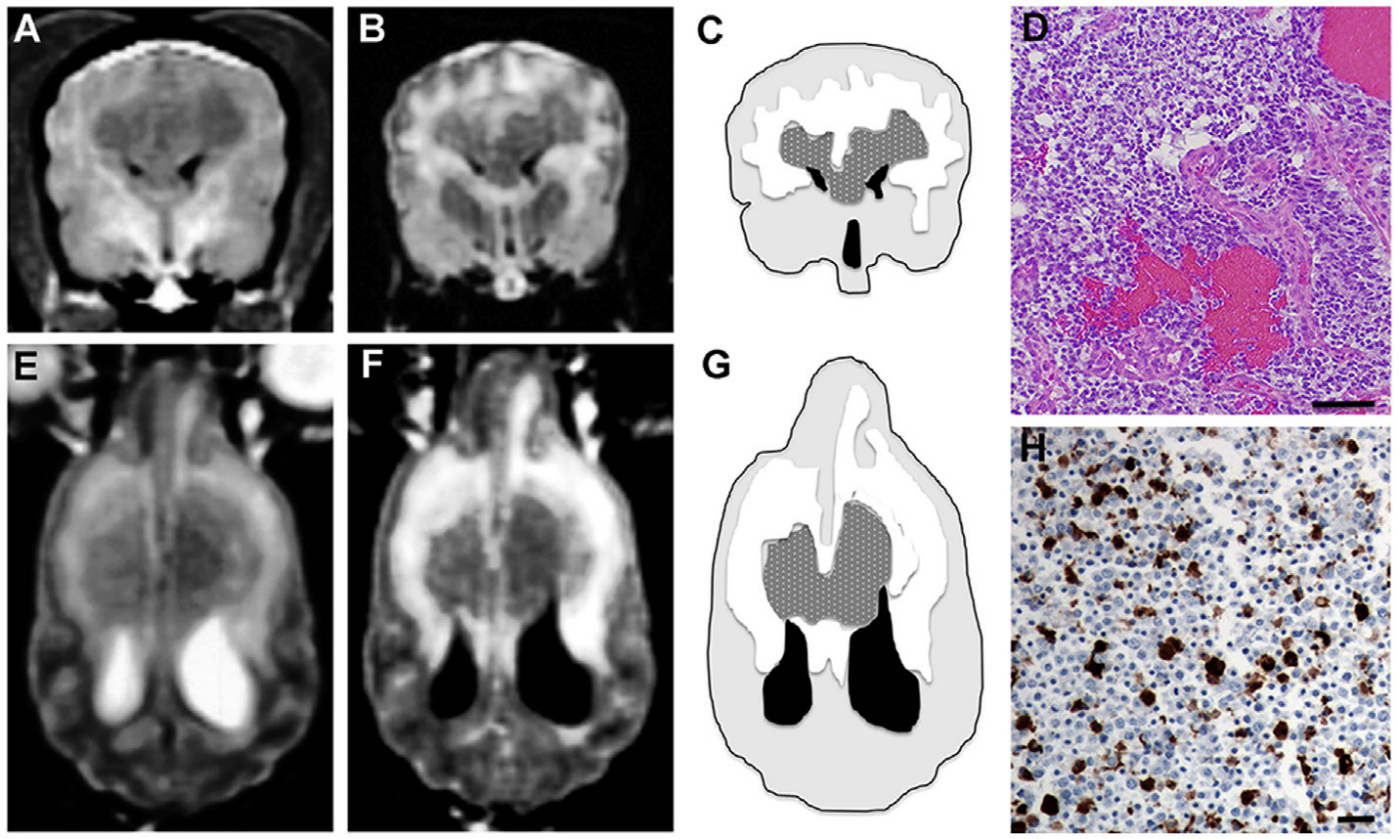
**Symmetrical butterfly glioblastoma, Case 3**. Transverse T1 **(A)** and FLAIR **(B)** MR images at the level of the cruciate gyrus, demonstrating an intra-axial, bilaterally symmetric mass lesion present in the frontoparietal regions. Schematic, MR intensity-segmented transverse **(C)** and dorsal planar **(G)** representations of symmetrical bihemispheric appearance of butterfly GBM (stippled gray), perilesional edema (white), brain parenchyma (light gray), and ventricles (black). Histopathological features of GBM **(D)** include marked hypercellularity, cellular pleomorphism, and microvascular proliferation. Oligodendroglial components of the tumor are present throughout the section (H&E stain, bar = 200 μm). In the dorsal planar PD-T2 **(E)** and FLAIR **(F)** images, the mass attenuates the rostral aspects of the lateral ventricles and is associated with extensive symmetrical perilesional hyperintensity within the surrounding white matter, consistent with edema. The mass demonstrates heterogeneous signal intensity in all sequences. **(H)** 50% of neoplastic astrocytes demonstrate intense GFAP staining, while neoplastic oligodendrocytes lack GFAP immunoreactivity (GFAP stain, horseradish peroxidase with 3,3′-diaminobenzidine substrate, bar = 50 μm).

Magnetic resonance imaging of the brain revealed a symmetrical, lobulated intra-axial mass involving the subcortical white matter of the frontoparietal regions of both cerebral hemispheres that extended across and connected the two cerebral lesions *via* the corpus callosum (Figure 4). The mass was predominantly T1 hypointense (Figure 4A), heterogeneously T2, and FLAIR iso- to hyperintense (Figures 4B,E,F) and compressed the rostral portions of the lateral ventricles. An extensive halo of T2 and FLAIR hyperintensity was present in the subcortical white matter surrounding the mass, consistent with perilesional edema (Figures 4C,G). No enhancement of the mass was observed after gadolinium administration.

The dog was treated with phenobarbital (3.2 mg/kg PO q 12 h) and prednisone (0.6 mg/kg PO q 12 h) and discharged. Eleven days later, the dog developed status epilepticus and died. On gross necropsy examination, the dorsal surface of the cerebrum appeared diffusely swollen. Transverse sectioning revealed a poorly defined, symmetrical, gray–tan intraparenchymal mass that was centered in the frontoparietal regions of both the cerebral hemispheres and was compressing the lateral ventricles ventrally. On midline, the mass infiltrated the falx cerebri. In its course adjacent to the mass, the dorsal sagittal sinus was thrombosed.

The corpus callosum was virtually effaced by continuous sheet of pleomorphic neoplastic astrocytes, with tumor cells extending throughout the white matter and obliterating the cerebrocortical architecture of both cerebral hemispheres. Regions of intratumoral necrosis, hemorrhage, and microvascular proliferation were present (Figure 4D). Several islands or cords of neoplastic oligodendroglial cells were found adjacent to necrohemorrhagic areas in the subcortical white matter. Patchy immunoreactivity to Olig-2 was found within the islands of cells with oligodendroglial morphology. Extensive neoplastic invasion of the meninges were noted in the parafalcine region. Subependymal tumor invasion was also noted in both lateral ventricles, and tumor cell nests were present within the left ventricular lumen. Immunoreactivity to GFAP was noted in 50% of neoplastic astrocytes (Figure 4H). Several distant and discrete leptomeningeal tumor foci that were 1–2 mm in diameter were noted in the left piriform and left ventral olfactory areas. The final diagnosis was bilateral cerebral glioblastoma with oligodendroglial component.

## Discussion

This series describes the MRI features associated with BG in three dogs. All the dogs reported here had tumors displaying classical morphological and immunohistochemical features of the GBM (BG-GBM) phenotype (8), which is also the most common tumor histology associated with the BG appearance in humans (1, 2). While patterns of canine GBM infiltration into the neural parenchyma mimic those seen in humans, with tumor spread occurring *via* white matter tracts, perivascular spaces, cerebrospinal fluid pathways, and subependymal routes, BG has not been an entity previously recognized in dogs (3–7).

In this series of dogs, BG was clinically characterized by the presence of neurological dysfunction referable to multifocal or diffuse forebrain disease, and neuroradiologically there were lesions in both the cerebral hemispheres. These features are atypical in dogs with supratentorial gliomas, in which the majority have a clinical presentation reflecting lateralized or historical, non-localizing (behavioral changes, seizures) forebrain disease, corresponding with a solitary intra-axial mass on MRI (4, 5). On MRI, canine BG appear as bihemispheric, intra-axial, mass lesions that predominantly affect the corpus callosum and subcortical white matter of the frontoparietal regions and are associated with extensive perilesional edema and mass effect. The presence of significant perilesional edema and mass effect can make detection of lesion extension into the corpus callosum difficult with MRI, especially in the absence of contrast enhancement, as seen in Cases 2 and 3. In the event multiple, discrete intraparenchymal or bihemispheric lesions with MRI characteristics of glioma are identified in the absence of involvement of the corpus callosum or evidence of other signal abnormalities connecting the lesions, consideration should be given to the possibility of the presence of a synchronous multicentric glioma or metastatic neoplasm (9). The intrinsic MRI signal characteristics of the BG cases reported here are similar to what has previously been described in canine GBM, with tumors being iso- to hypointense on T1 images, heterogeneously iso- to hyperintense on T2, and FLAIR sequences, and the majority demonstrating some degree of contrast enhancement (3–7). Notably, the 2/3 enhancing canine BG reported here demonstrated moderate to marked diffuse mass enhancement, which differs from the peripheral ring-enhancing pattern that is typically observed in human BG-GBM (1, 2, 9).

There were variable degrees of lesion symmetry seen in the BG of these dogs, ranging from the GBM of Case 3, which displayed both interhemispheric uniformity in the anatomic distribution of the lesion as well as symmetrical intra- and perilesional signal changes, to the highly asymmetric tumor burden between cerebral hemispheres noted in Case 1. Despite the common inclusion of the “symmetric” term in the definition of BG, a recent study demonstrated that only 4/23 of human BG were symmetrical, with symmetry defined as having <10% difference in the contrast-enhancing tumor burden between cerebral hemispheres (2). Given that human and canine BG-GBM can have an asymmetrical appearance, and that BG-GBM in both species (Case 3) may be non-enhancing, we propose an inclusive modification to the operational definition of BG introduced by Dziurzynski and colleagues and suggest that BG be defined as a high-grade glioma crossing the corpus callosum, resulting in a contiguous tumor burden involving both cerebral hemispheres at initial presentation (1, 2).

In humans, the corpus callosum is resistant to the spread of edema and infection, and, thus, there is a limited list of imaging differentials for mass lesions infiltrating this structure (1). These differential diagnoses include BG, demyelinating butterfly pseudoglioma, multiple sclerosis, lymphoma, brain metastases, protozoal encephalitis, and neuronal ceroid lipofuscinosis (10). Given the MRI features of the lesions in this series, we also considered variants of meningoencephalitis of unknown origin, such as necrotizing leukoencephalitis and granulomatous meningoencephalitis, as potential differentials. Type IIC gliomatosis cerebri (GC) is another diagnostic consideration for a focal glial neoplasm accompanied by diffuse extension of the tumor within the central nervous system. However, unlike the cases described here, dogs reported to date with Type IIC GC had oligodendrogliocytic tumors, no MRI evidence of disease extension beyond the focal lesion, and the majority had widespread microscopic disease at necropsy (11). In contrast, in this case series, the neuropathological extension of GBM correlated well with the topography of lesions observed on MRI, and the tumors were universally associated with a significant disruption of tissue architecture in both cerebral hemispheres.

Although rare, BG should be considered as a differential diagnosis in dogs with intra-axial mass lesions with inherent signal changes consistent with glioma that extend through the corpus callosum to involve both the cerebral hemispheres. In both dogs and humans, the neuroradiological appearance of BG may often be asymmetrical and is frequently caused by GBM.

## Author Contributions

All authors meet the criteria for authorship. JHR, TP, and SE participated in clinical case management. JHR, KC, and WD drafted the manuscript. KC reviewed the neuroimaging studies, and JLR provided the neuropathological interpretations. JHR and WD provided material support for performance of the immunohistochemical studies. All authors participated in the review and the editing of the manuscript.

## Conflict of Interest Statement

The authors declare that the research was conducted in the absence of any commercial or financial relationships that could be construed as a potential conflict of interest.

## Abbreviations

BG, butterfly glioma; FLAIR, fluid-attenuated inversion recovery; GBM, glioblastoma mutliforme (WHO Grade IV astrocytoma); GC, gliomatosis cerebri; IV, intravenous; MRI, magnetic resonance imaging; SACS, small animal coma scale; WHO, World Health Organization.
