# Supplementary material for: Canine Butterfly Glioblastomas: A Neuroradiological Review
Source: Front Vet Sci. 2016 May 19;3:40. doi: 10.3389/fvets.2016.00040 (PMC4931820; doi:10.3389/fvets.2016.00040)
Supplement: Supplementary file 1 [file data_sheet_1.docx]

**CANINE BUTTERFLY GLIOBLASTOMAS: A NEURORADIOLOGICAL REVIEW**

John H. Rossmeisl,^*^ Kemba Clapp, Theresa E. Pancotto, Samantha Emch, John L. Robertson, Waldemar Debinski

***Correspondence:** John H. Rossmeisl; jrossme@vt.edu

# Supplementary Data

**Magnetic Resonance Imaging (MRI) and Neuropathological Examination Methods**

MRI examinations of the head were performed under general anesthesia in all cases. Various matrix sizes and fields of view were used. Case 1 was imaged with a 1.5T superconducting magnet (Philips Intera, Philips Healthcare, Andover, MA, USA). The following sequences were obtained: pre- and post-contrast fast spin-echo T1-weighted (T1W-FSE) in three planes, T2-weighted (T2W-FSE) in the sagittal and transverse planes, gradient-echo T2-weighted (T2W-GRE) and fluid attenuated inversion recovery (FLAIR) images in the transverse plane, and diffusion weighted images (DWI) obtained using an echo planar technique and *b* value of 1000. Proprietary software was used to construct apparent diffusion coefficient maps from DWI images.

Case 2 was imaged with a 0.2T system using a multipurpose flex coil (Esaote Vet-MR, Genoa, Italy), and Case 3 with a 0.5T system (Signa Contour, General Electric Healthcare, Chicago, IL, USA) using a dedicated head coil. In cases 2 and 3, the following sequences obtained: spin-echo T1-weighted (T1W-SE) images in the sagittal and transverse planes, spin-echo T2-weighted (T2W-SE) images in the sagittal and transverse planes, spin-echo proton density T2-weighted (T2W-PD) in the dorsal plane, and gradient-echo T2-weighted (T2W-GRE) images in the transverse plane. Post-contrast T1W-SE images were also obtained in the dorsal, transverse, and sagittal planes. In all dogs, post-contrast T1W images were obtained following intravenous injection with 0.1 mmol/kg of gadopentetate dimeglumine (Magnevist, Bayer, Wayne, NJ, USA).

For histopathological analyses, brain tissues were immersion fixed in 10% neutral buffered formalin and embedded in paraffin-polymer. Microscopic examinations were performed on 5-μm thick sections stained with hematoxylin and eosin (H&E). Immunohistochemical staining was performed on selected tissues from each case with antibodies to glial fibrillary acidic protein (GFAP; M0761, clone 6F2, 1:150 dilution; Dako, Carpinteria, CA, USA), vimentin (Clone V9, Dako, 1:300), and Olig-2 (Millipore, polyclonal 1:100, Billerica, MA, USA) using previously described methods.^a^

^a^Rossmeisl JH, Pineyro P, Sponenberg DP, Garman RH, Jortner BS. Clinicopathologic features of intracranial central neurocytomas in 2 dogs. *J Vet Int Med* (2012) 26: 186-191. doi: 10.1111/j.1939-1676.2011.00862.x

**
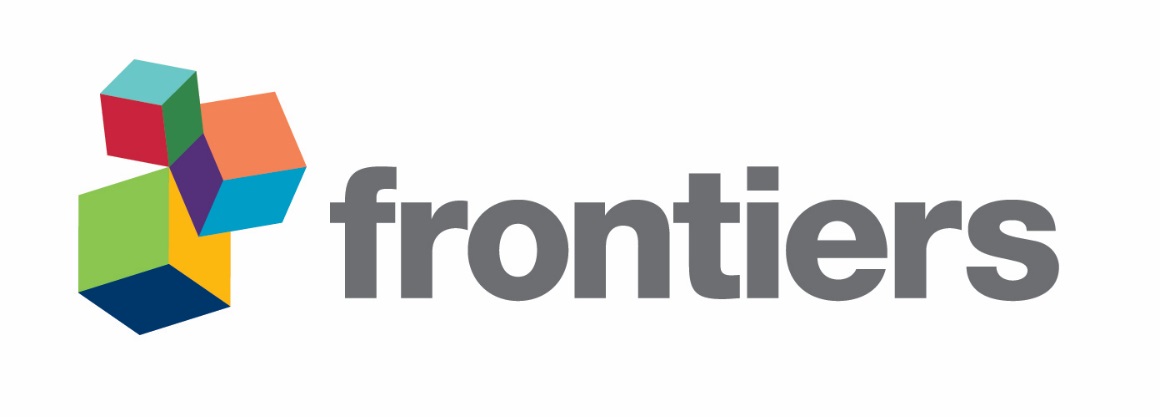
**
